# Bile acids as a predictor for all-cause mortality in peritoneal dialysis patients: a retrospective cohort study

**DOI:** 10.1186/s12882-026-05187-3

**Published:** 2026-07-10

**Authors:** Siyuan Zhao, Xiaoxiao Wang, Qiong Bai, Wen Tang

**Affiliations:** 1 Department of Nephrology, Peking University Third Hospital, 49 North Garden Rd, Haidian District, 100191 Beijing, P. R. China; 2 Research Center of Clinical Epidemiology, Peking University Third Hospital, Beijing, China

**Keywords:** Bile acids, Peritoneal dialysis, Mortality

## Abstract

**Background:**

Bile acids (BAs) are now recognized as endocrine hormones involved in glucose and lipid metabolism regulation and inflammation control. Recent studies have also shown the association of BAs level in chronic kidney disease progression and hemodialysis patients’ prognosis. However, their role in peritoneal dialysis (PD) is unknown. Therefore, based on a longitudinal cohort, this study aims to explore the relationship of BAs and all-cause mortality in PD patients.

**Methods:**

A retrospective cohort study was conducted involving 517 PD patients. Serum BAs level prior to PD catheterization were collected. Restricted cubic spline (RCS) analysis with full adjustment was used to characterize the dose-response relationship between serum BAs and all-cause mortality. Cox proportional hazards regression models were performed to assess the independent association between BAs levels and all-cause mortality.

**Results:**

A total of 517 patients were included. During a median follow-up of 3.65 (1.36, 5.46) years, death occurred in 210 of 517 (40.6%) patients. The relationship between BAs levels and the risk of all-cause mortality was fitted using multivariate RCS adjusted for clinical relevant factors showing that BAs levels were linearly associated with all-cause mortality (p for non-linearity = 0.589). Further Kaplan-Meier survival analysis revealed a significantly higher all-cause mortality risk in the high BAs group (log rank score 13.04, *p* < 0.001). Cox regression showed that elevated BAs levels were an independent risk factor for all-cause mortality in PD patients (HR = 1.056, 95% CI: 1.005–1.109, *p* = 0.031) after adjusting for confounding factors.

**Conclusion:**

Elevated pre-dialysis serum BAs levels are independently associated with increased all-cause mortality in PD patients. Serum BAs may represent a potential prognostic indicator, although further validation is required before clinical application.

## Introduction

Peritoneal dialysis (PD) accounts for 11% of all dialysis treatments worldwide [1, 2]. Long-term PD use is associated with glucose and lipid metabolic abnormalities, which contribute to adverse clinical outcome [3, 4]. Bile acids (BAs), traditionally known for their role in lipid digestion, serve as sensitive indicators of hepatocyte function and integrity, and are used to assess cholestasis [5]. They are now recognized as endocrine hormones involved in glucose and lipid metabolism regulation and inflammation control [6–8]. BAs could improve insulin sensitivity and promote high density lipoprotein function while reducing very low-density lipoprotein (VLDL) levels. These metabolic benefits support its investigation as a potential plasma biomarker. Recent studies have also shown the association of BAs with Chronic Kidney Disease (CKD) disease progression and clinical prognosis [9–11].

However, the association of altered BAs levels and outcome of dialysis patients is unclear. A recent study in hemodialysis (HD) patients demonstrated that lower BAs levels were associated with lower lipid levels in patients with diabetes mellitus (DM) on HD and could be an independent risk factor for all-cause death in patients with DM on HD [9]. The observed correlation warrants further investigation into the role of BAs in PD outcome. We chose to measure total serum BAs because it represents a routine, widely accessible, and cost-effective clinical biomarker for real-world prognostic evaluation. In contrast, individual BA subtypes require specialized metabolomic platforms that are not routinely available in clinical practice for risk stratification of PD patients.

Therefore, in the present study, based on a longitudinal cohort, this study aims to explore the relationship of BAs and all-cause mortality in PD patients.

## Methods

### Study population

In this retrospective cohort study, all incident patients who started PD therapy from 1 July 2010 to 12 April 2024 from Peking University Third Hospital were included. Patients were excluded if they had age less than seventeen years or if they had malignant tumor, hepatobiliary diseases (active hepatitis, liver cirrhosis and cholecystolithiasis), or without pre-dialysis BAs values. Patients were followed until death, cessation of PD or end of study as of 25 September 2024.

In our center, pre-dialysis patients need to be hospitalized to department of nephrology for PD catheter implantation. Therefore, patients’ detailed comorbidities, demographics, laboratory parameters were recorded when admitted. During the study period, patients’ hospitalization electronic medical charts were carefully reviewed to obtain the data, Serum BAs level prior to PD catheterization were collected. Serum BAs were measured using a standardized enzymatic colorimetric assay on a fully automated biochemical analyzer. Other assay indictors including hemoglobin, albumin, creatinine, calcium, phosphorus, total cholesterol, triglycerides, high-density lipoprotein cholesterol (HDL-c) and low-density lipoprotein cholesterol (LDL-c) were also obtained. All blood samples were collected after an overnight fasting of at least 8 h to ensure standardization and consistency of BAs levels. The estimated glomerular filtration rate (eGFR) was estimated by Chronic Kidney Disease Epidemiology Collaboration (CKD-EPI) creatinine equation [12]. All baseline data, including serum BAs and other laboratory parameters, were collected at admission and prior to PD catheter insertion and PD initiation. Therefore, PD‑related parameters such as Kt/V, peritoneal transport status, peritonitis rate, and residual kidney function during PD treatment were not available at baseline and could not be included in the primary analysis.

The primary outcomes examined were all-cause mortality on PD (censored for loss to follow-up, renal transplantation, recovery of renal function, transferred to HD, transfer to other dialysis centers and end of study).

This study has been approved by the Ethics Committee of Peking University Third Hospital (No. 096 − 03). The research protocol conforms to the ethical standards of the 1964 Declaration of Helsinki and its later amendments.

### Statistical analysis

Results were expressed as frequencies and percentages for categorical variables, mean ± standard deviation for continuous normally distributed variables, and median (interquartile range, IQR) for continuous variables that were not normally distributed. Comparison for patients in different groups was performed using chi-square tests, two-tailed unpaired t-tests or Mann-Whitney’s tests, depending on data distribution. Missing values for baseline variables were less than 5% and regarded as negligible. No imputation was performed for missing data, and all statistical analyses were conducted using the available complete case data only.

Restricted cubic spline (RCS) curve with 3 knots was displayed to test nonlinearity when estimating all-cause mortality [13].We constructed multivariable RCS models by a full adjusted model. A likelihood ratio test was performed to compare the model with a linear term to the model with the full spline term. During the likelihood ratio test, if BAs levels were linearly associated with all-cause mortality (*p* > 0.05), BAs level is incorporated as continuous variables in multivariable Cox regression models, otherwise, they would be utilized as categorical variable in the multivariable Cox regression model. Kaplan-Meier survival curves were used to evaluate the survival curves of all-cause mortality, and the Log-rank test was applied for the comparison. For the multivariable Cox proportional hazards regression model, covariates were selected based on clinical relevance as established prognostic factors in PD patients and variables with *p* < 0.05 in univariate analysis. The proportional hazards assumption was verified, and multicollinearity was assessed using the variance inflation factor (VIF). A competing risk analysis was performed using the Fine–Gray subdistribution hazards model to evaluate the association between baseline serum BAs and all-cause mortality. Transfer to hemodialysis and renal transplantation were treated as competing events. Subdistribution hazard ratios (SHRs) with 95% confidence intervals (CIs) were estimated.

Statistical analysis was performed using IBM SPSS software, version 22.0 (Armonk, NY) and R (version 4.1.3). A *p*-value < 0.05 was considered statistically significant.

## Results

### Baseline clinical characteristics of the study population and all-cause mortality

**Fig. 1:**
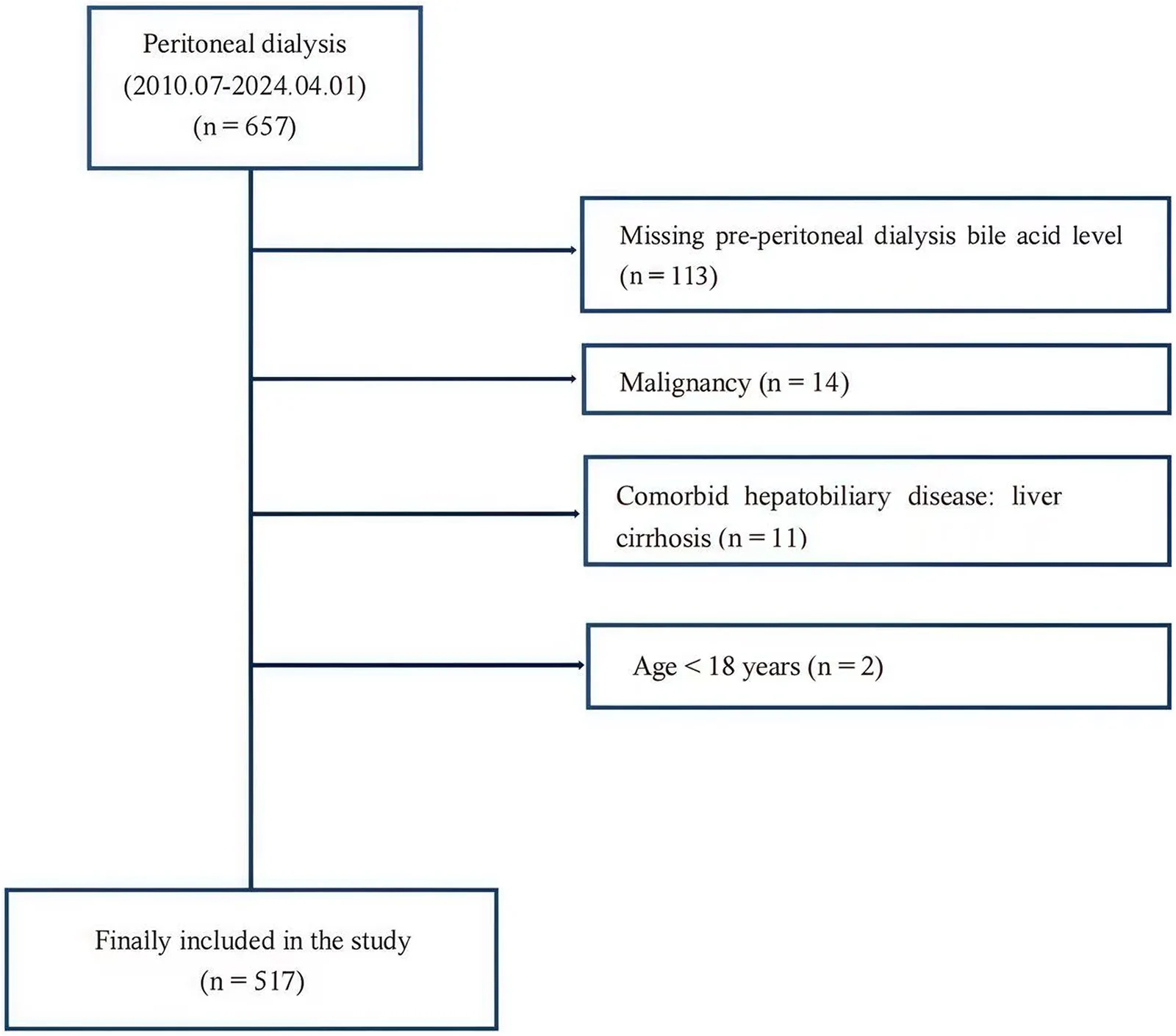
Flowchart of this study

A total of 517 patients were included in the study (Fig. 1). During a median follow-up of 3.65 (1.36, 5.46) years, death occurred in 210 of 517 (40.6%) PD patients. 38 patients (7.4%) transferred to HD, 46 patients (8.9%) underwent renal transplantation. Loss to follow up occurred in 26 patients (5.0%) and 34 patients (6.6%) transferred to other dialysis centers.

All 517 PD patients were stratified into two groups according to the median level of BAs: low BAs group (BAs ≤ 2.20µmol/L) and high BAs group (BAs > 2.20µmol/L). The baseline clinical characteristics across the two BAs subgroups are summarized in Table 1. Compared with the low BAs group, patients in the high BAs group had significantly lower levels of triglycerides (*p* < 0.001), total cholesterol (*p* = 0.012), LDL-c (*p* = 0.012), and serum phosphorus (*p* = 0.003), along with a notably higher eGFR (*p* = 0.033). With respect to comorbidity profiles, the high BAs group presented with a higher prevalence of coronary heart disease (CHD) (*p* = 0.008) yet a lower prevalence of hypertension (*p* = 0.016) relative to the low BAs group. There were no substantial missing data for key baseline variables, and complete case analysis was applied without data imputation.

**Table 1:** Baseline clinical characteristic of all patients at the initiation of PD

**Variables**	**All**(n=517)	**Low BAs**(n=262)	**High BAs**(n=255)	***p*** **-value**
Age, years	60.9 (46.7-73.6)	56.9 (45.4-70.2)	64.4 (49.8-75.4)	0.002
Male, n (%)	301 (58.2)	141 (53.8)	160 (62.7)	0.049
BAs, µmol/L	2.20 (1.40-3.60)	1.40 (1.00-1.80)	3.60 (2.85-5.05)	<0.001
BMI, kg/m^2^	23.51 (21.22-25.94)	23.33 (21.23-26.14)	23.67 (21.22-25.85)	0.933
Hypertension, n (%)	353 (68.3)	191 (72.9)	162 (63.5)	0.016
CHD, n (%)	125 (24.2)	50 (19.1)	75 (29.4)	0.008
CVD, n (%)	75 (14.5)	32 (12.2)	43 (16.9)	0.180
DM, n (%)	287 (55.5)	145 (55.3)	142 (55.7)	1.000
eGFR, ml/min/1.73m^2^	4.76 (3.82-6.08)	4.64 (3.67-5.92)	4.96 (3.90-6.24)	0.033
Potassium, mmol/L	4.52 (4.04-5.02)	4.56 (4.12-5.10)	4.48 (3.95-4.98)	0.055
Sodium, mmol/L	139.00 (137.00-141.00)	139.00 (137.00-141.00)	139.00 (137.00-141.00)	0.289
Serum albumin, g/L	36.28 ± 5.36	36.11 ± 5.39 (32.60-40.00)	36.45 ± 5.18(32.80-40.00)	0.462
Hemoglobin, g/L	88.00 (75.00-101.00)	87.00 (73.00-100.00)	89.00 (76.00-101.00)	0.442
Triglyceride, mmol/L	1.34 (1.01-1.98)	1.46 (1.11-2.07)	1.23 (0.88-1.75)	<0.001
Total cholesterol, mmol/L	4.04 (3.42-4.98)	4.22 (3.51-5.24)	3.96 (3.32-4.75)	0.012
LDL-c, mmol/L	2.36 (1.80-3.09)	2.41 (1.87-3.25)	2.29 (1.73-2.93)	0.012
HDL-c, mmol/L	0.93 (0.78-1.18)	0.93 (0.78-1.18)	0.94 (0.79-1.18)	0.735
Serum calcium, mmol/L	2.04 (1.86-2.20)	2.04 (1.81-2.20)	2.05 (1.88-2.20)	0.352
Serum phosphorus, mmol/L	1.94 (1.55-2.36)	1.98 (1.65-2.47)	1.88 (1.50-2.24)	0.003
Death, n (%)	210 (40.6)	93 (35.4)	117 (45.9)	0.021

### The relationship between BAs levels and risk of all-cause mortality

**Fig. 2:**
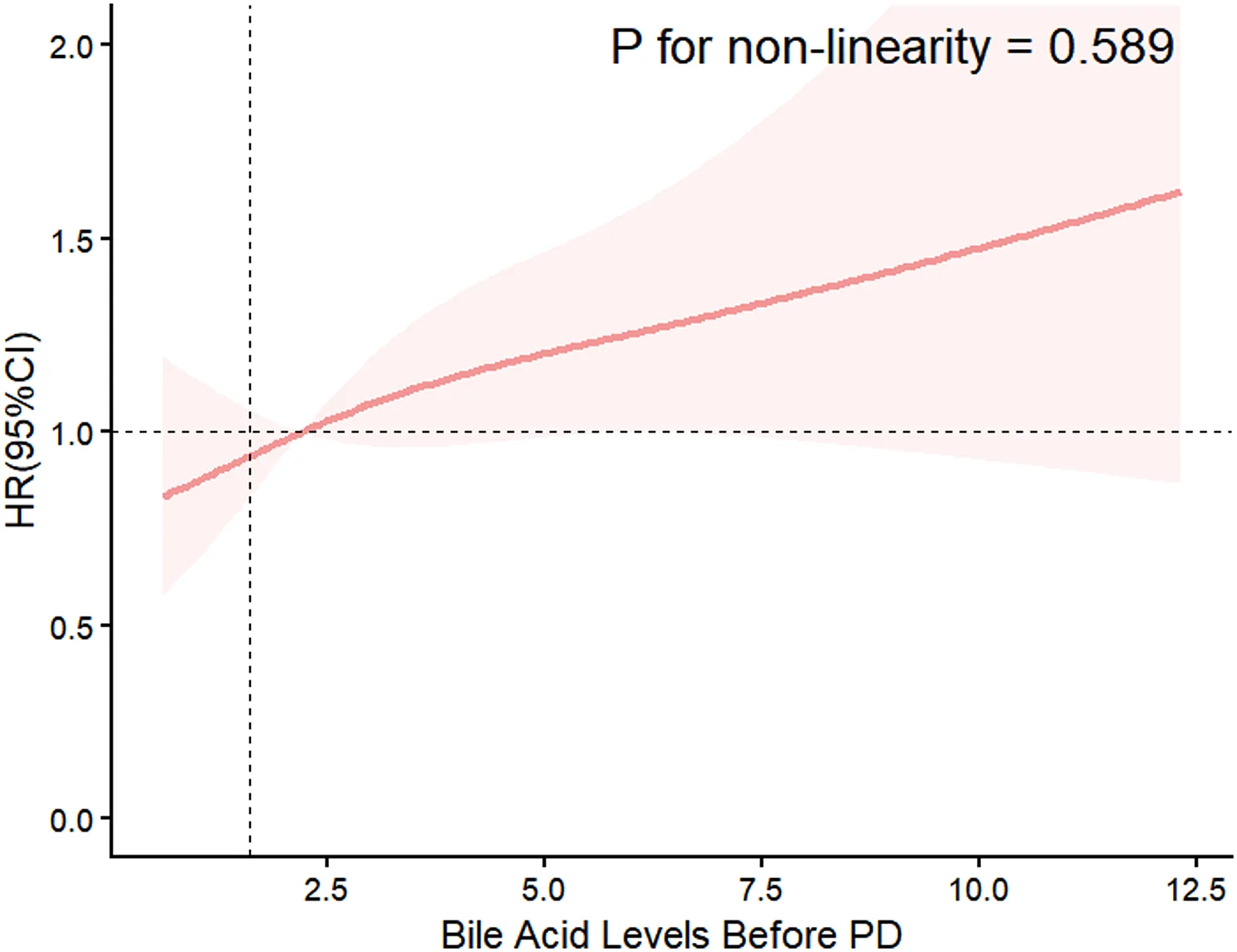
The association between bile acids and all-cause mortality risk after full adjustment. Clinically relevant variables were included in multivariate RCS analysis, including age, sex, eGFR, hemoglobin, blood glucose, serum phosphorus, albumin, hypertension, coronary heart disease, cerebrovascular disease, and diabetes mellitus

The relationship between BAs levels and the risk of all-cause mortality was fitted using multivariate RCS analysis adjusted for age, gender, DM, CHD, hypertension, cerebrovascular disease (CVD), eGFR, hemoglobin, blood glucose, serum phosphorus and serum albumin. Consequently, BAs levels were linearly and positively associated with all-cause mortality (p for non-linear = 0.589) (Fig. 2). The risk of all-cause mortality increased progressively with rising serum BAs levels, which supported the use of BAs as a continuous variable in subsequent Cox regression models.

**Fig. 3:**
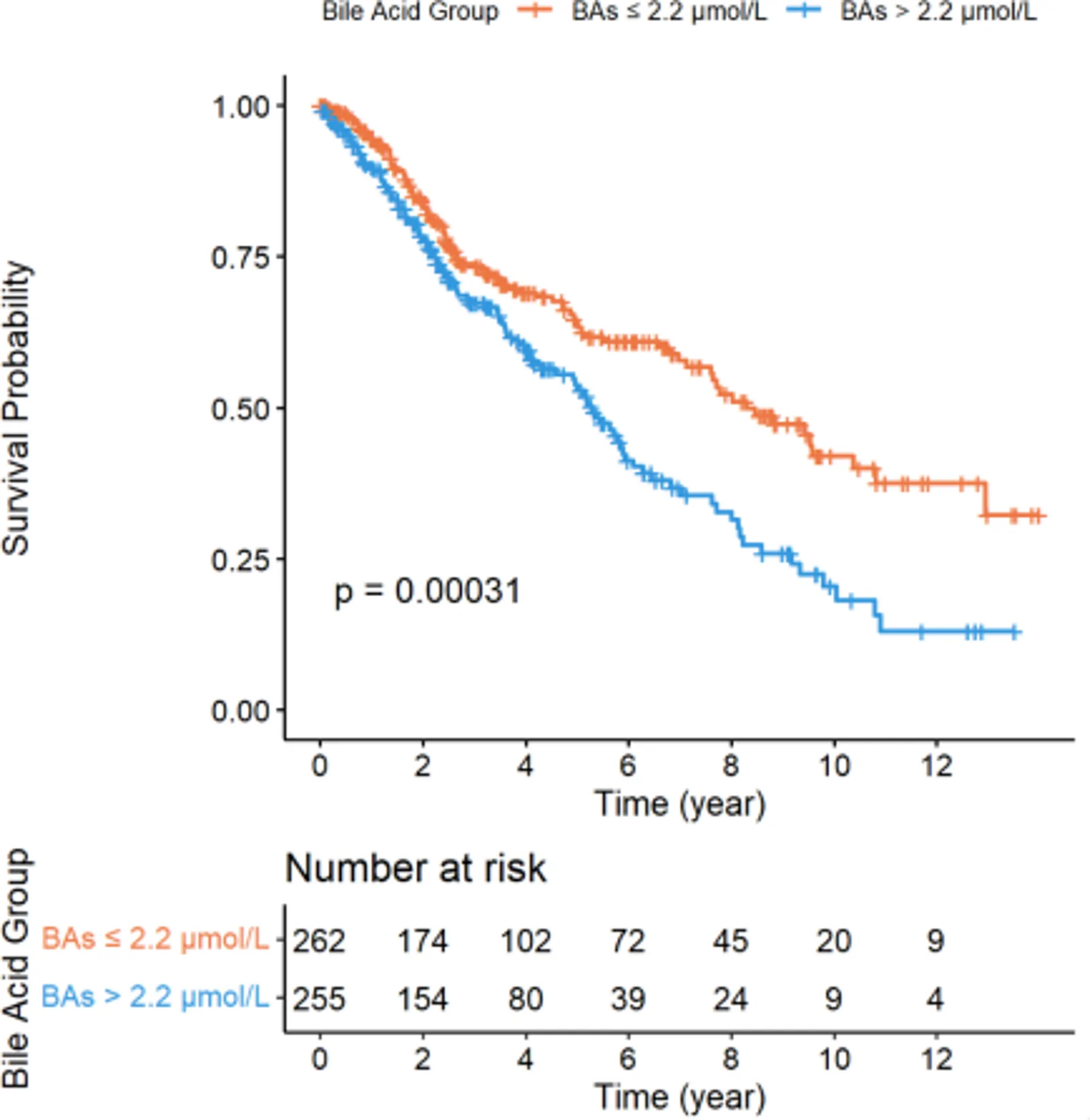
Kaplan-Meier survival curve for all-cause mortality comparing PD patients with different levels of Bile acids (BAs)

Kaplan-Meier survival analysis demonstrated that patients in the high BAs group had a significantly lower cumulative survival rate compared with those in the low BAs group. The log-rank test confirmed a marked difference between the two groups (log-rank score = 13.04, *P* < 0.001) (Fig. 3), indicating that higher baseline BAs levels were associated with worse long-term survival in PD patients.

Univariate Cox regression analysis identified that BAs levels, age, hypertension, hemoglobin, serum albumin, serum phosphorus, serum potassium levels, CHD, diabetes, and CVD were all significantly associated with all-cause mortality (all *p* < 0.05). In the multivariate Cox proportional hazards regression model, elevated BAs levels discovered to be an independent factor for all-cause mortality in PD patients (HR = 1.056, 95% CI: 1.005–1.109, *p* = 0.031) after adjusting for age, serum albumin, CHD, CVD, eGFR, hemoglobin, serum phosphorus, hypertension, and DM. After accounting for competing risks of transferred to hemodialysis and transplantation, higher BA levels demonstrated a significant association with increased all-cause mortality (SHR = 1.08, 95% CI: 1.03–1.12, *p* < 0.001). Given the narrow physiological range of serum BAs, this per-unit HR appears modest. To improve clinical interpretability, we further stratified patients by the median BAs level (≤ 2.20 vs. > 2.20 µmol/L). Compared with the low BAs group, the high BAs group had a higher crude mortality rate (45.9% vs. 35.4%), with an absolute risk difference (ARD) of 10.5%, indicating a clinically meaningful prognostic impact. Details are presented in Table 2.

**Table 2:** Cox regression analysis for all-cause mortality in PD patients

Variables		All (*n* = 517)	Low BAs (*n* = 262)		High BAs (*n* = 255)			*p*-value
Age, years		60.9 (46.7–73.6)	56.9 (45.4–70.2)			64.4 (49.8–75.4)		0.002
Male, n (%)		301 (58.2)	141 (53.8)			160 (62.7)		0.049
BAs, µmol/L		2.20 (1.40–3.60)	1.40 (1.00-1.80)			3.60 (2.85–5.05)		< 0.001
BMI, kg/m^2^		23.51 (21.22–25.94)	23.33 (21.23–26.14)			23.67 (21.22–25.85)		0.933
Hypertension, n (%)		353 (68.3)	191 (72.9)			162 (63.5)		0.016
CHD, n (%)		125 (24.2)	50 (19.1)			75 (29.4)		0.008
CVD, n (%)		75 (14.5)	32 (12.2)			43 (16.9)		0.180
DM, n (%)		287 (55.5)	145 (55.3)			142 (55.7)		1.000
eGFR, ml/min/1.73m^2^		4.76 (3.82–6.08)	4.64 (3.67–5.92)			4.96 (3.90–6.24)		0.033
Potassium, mmol/L		4.52 (4.04–5.02)	4.56 (4.12–5.10)			4.48 (3.95–4.98)		0.055
Sodium, mmol/L		139.00 (137.00-141.00)	139.00 (137.00-141.00)			139.00 (137.00-141.00)		0.289
Serum albumin, g/L		36.28 ± 5.36	36.11 ± 5.39 (32.60–40.00)			36.45 ± 5.18(32.80–40.00)		0.462
Hemoglobin, g/L		88.00 (75.00-101.00)	87.00 (73.00-100.00)			89.00 (76.00-101.00)		0.442
Triglyceride, mmol/L		1.34 (1.01–1.98)	1.46 (1.11–2.07)			1.23 (0.88–1.75)		< 0.001
Total cholesterol, mmol/L		4.04 (3.42–4.98)	4.22 (3.51–5.24)			3.96 (3.32–4.75)		0.012
LDL-c, mmol/L		2.36 (1.80–3.09)	2.41 (1.87–3.25)			2.29 (1.73–2.93)		0.012
HDL-c, mmol/L		0.93 (0.78–1.18)	0.93 (0.78–1.18)			0.94 (0.79–1.18)		0.735
Serum calcium, mmol/L		2.04 (1.86–2.20)	2.04 (1.81–2.20)			2.05 (1.88–2.20)		0.352
Serum phosphorus, mmol/L		1.94 (1.55–2.36)	1.98 (1.65–2.47)			1.88 (1.50–2.24)		0.003
Death, n (%)		210 (40.6)	93 (35.4)			117 (45.9)		0.021

Abbreviations: CI, confidence interval; HR, hazard ratio; BMI, body mass index; BAs, bile acids; LDL-c, low-density lipoprotein cholesterol; HDL-c, high-density lipoprotein cholesterol; CHD, coronary artery disease; CVD, cerebrovascular disease; eGFR, estimated glomerular filtration rate

For the multivariable Cox proportional hazards regression model, covariates were selected based on clinical relevance as established prognostic factors in PD patients and variables with *p* < 0.05 in univariate analysis. The proportional hazards assumption was verified, and multicollinearity was assessed using the variance inflation factor (VIF)

## Discussion

In this retrospective cohort study with a large sample size and long-term follow-up, we provide evidence for an association between serum BAs levels and all-cause mortality in PD patients. Further analyses confirmed a linear relationship between BAs levels and mortality risk. Elevated serum BAs were identified as an independent risk factor for all-cause mortality in PD patients, and this association remained robust after adjustment for established prognostic variables.

In addition to higher all-cause mortality observed in the high BAs group, several baseline characteristics is observed differed between the low and high BAs groups. Patients with elevated BAs levels were older, exhibited a greater prevalence of underlying coronary heart disease, and higher eGFR; conversely, they had a lower prevalence of hypertension, lower total cholesterol, lower LDL-C, and lower serum phosphorus levels compared with those in the low BAs group. Notably, BAs remained an independent predictor of all-cause mortality after multivariable adjustment for lipids, age, comorbidities, and renal function. This indicates that the BAs-mortality association is not explained by lipid differences or other baseline factors. Consistent with existing evidence, BAs may influence prognosis through non-lipid pathways, including inflammation, oxidative stress, and signaling involved in cardiovascular and renal fibrosis.

BAs act not only as lipid emulsifiers but also as pleiotropic signaling molecules that regulate glucose and lipid metabolism via farnesoid X receptor (FXR) and G-protein-coupled bile acid receptor (TGR5) [14–18]. Dysregulated BA signaling disrupts metabolic homeostasis, promoting insulin resistance and metabolic dysfunction [19, 20]. In PD patients, elevated BAs may increase mortality risk through three key mechanisms. First, high BAs alter gut microbiota composition and impair intestinal immunity, exacerbating chronic inflammation [21, 22]. Second, dysregulated BA signaling disrupts glucose and lipid metabolism, increasing cardiovascular risk [23]. Third, excess hydrophobic BAs exert direct cytotoxicity, worsening tissue injury and chronic damage in dialysis patients.

Research investigating the prognostic impact of BAs on PD patients remains remarkably scarce in the existing literature. Only a study employing non-targeted metabolomics indicated that increased levels of cholic acid and adenine, along with decreased isoleucine, were identified as independent risk factors for poor prognosis in PD patients, suggesting that levels of cholic acid, a BAs subtype, are positively associated with the risk of all-cause mortality in PD patients [10]. In the present study, with a specific focus on circulating BAs levels, we revealed that serum BAs levels are positively associated with PD patients’ all-cause mortality. Notably, this biomarker is characterized by its ready measurability in clinical settings, which lends greater feasibility to its potential application as a prognostic indicator for PD patients.

While our findings highlight the prognostic role of BAs in PD patients, studies in other populations, including those with diabetic nephropathy and end‑stage kidney disease (ESKD), have yielded inconsistent results. Notably, our findings differ markedly from those in HD cohorts, where lower BAs were associated with higher mortality [9]. Moreover, several studies have reported that elevated BAs, particularly deoxycholic acid, predict adverse outcomes in ESKD patients [24, 25]. These discrepancies may be explained by key differences in BAs handling between dialysis modalities. BAs are highly protein‑bound; HD relies on diffusion and poorly clears protein‑bound solutes, whereas PD enables continuous, prolonged clearance of such molecules, leading to distinct systemic BAs exposure and biological effects [26]. Additionally, prior HD studies enrolled maintenance patients, while our cohort included incident PD patients with baseline BAs measured pre‑dialysis. These differences in patient population and timing of measurement further contribute to divergent findings. Further research is needed to validate these mechanisms and clarify the differential prognostic impact of BAs across dialysis modalities.

Notably, patients with higher serum BAs had significantly lower triglycerides, total cholesterol, and LDL‑c than those with lower BAs. This inverse association indicates that elevated BAs in PD patients reflect not only cholestasis but also a distinct metabolic phenotype with reduced lipemia. BAs are synthesized from cholesterol and, beyond facilitating fat absorption, act as signaling molecules that suppress hepatic lipogenesis and promote cholesterol excretion via FXR‑mediated pathways [27, 28]. In CKD, elevated BAs may further lower circulating lipids by altering gut microbiota [29], impairing lipid absorption, and exacerbating inflammation‑related hepatic lipoprotein suppression. Importantly, after multivariable adjustment including lipid parameters, elevated BAs remained independently associated with increased all‑cause mortality. This indicates that lipids are unlikely to confound the BAs–mortality relationship, and that BAs may affect prognosis through non‑lipid pathways, such as chronic inflammation, gut dysbiosis, or direct cytotoxicity [30, 31].

This study is subject to several limitations that warrant acknowledgment. First, serum BAs levels were measured only at a single time point prior to PD initiation, which may not fully represent long-term BAs exposure during the entire follow-up period. Second, only total serum BAs were measured, whereas individual BAs subtypes are known to exert distinct, even opposing, biological effects on metabolism, inflammation, and cellular function. Failure to analyze specific BAs subtypes limits mechanistic interpretation and may mask divergent prognostic impacts of individual BAs species. Third, it is a single-center retrospective study, limiting generalizability and leaving potential residual confounding. Fourth, the observational design precludes causal inference. Additionally, key PD-related parameters (Kt/V, peritoneal transport, peritonitis, residual kidney function) were unavailable for adjustment. Future multicenter, prospective studies with serial BAs measurements and detailed PD parameters are needed to validate our findings.

In conclusion, in this retrospective cohort study, elevated pre-dialysis serum BAs levels were independently associated with an increased risk of all-cause mortality in PD patients. These findings suggest that serum BAs may serve as a potential prognostic indicator, although further validation in multicenter and prospective cohorts is required before strong clinical application can be established.

## Data Availability

The datasets used and/or analysed during the current study are available from the corresponding author on reasonable request.

## Abbreviations

BAs: Bile acids
CKD: Chronic kidney disease
CKD‑EPI: Chronic Kidney Disease Epidemiology Collaboration
CVD: Cerebrovascular disease
CHD: Coronary heart disease
DM: Diabetes mellitus
ESKD: End‑stage kidney disease
eGFR: Estimated glomerular filtration rate
FXR: Farnesoid X receptor
TGR5: G‑protein‑coupled bile acid receptor
HD: Hemodialysis
HDL‑c: High‑density lipoprotein cholesterol
IQR: Interquartile range
LDL‑c: Low‑density lipoprotein cholesterol
PD: Peritoneal dialysis
RCS: Restricted cubic spline
VIF: Variance inflation factor
VLDL: Very low‑density lipoprotein

## References

[1] Gu W, et al. Metabolic Syndrome and Mortality in Continuous Ambulatory Peritoneal Dialysis Patients: A 5-Year Prospective Cohort Study. Kidney Blood Press Res. 2019;44(5):1026–35. 10.1159/000502145 31522168

[2] Cho Y, et al. Peritoneal Dialysis Use and Practice Patterns: An International Survey Study. Am J Kidney Dis. 2021;77(3):315–25. 10.1053/j.ajkd.2020.05.032 32800844

[3] Wen Y, et al. High glucose concentrations in peritoneal dialysate are associated with all-cause and cardiovascular disease mortality in continuous ambulatory peritoneal dialysis patients. Perit Dial Int. 2015;35(1):70–7. 10.3747/pdi.2013.00083 PMC4335930 24293666

[4] Hsu KM, et al. Triglyceride-glucose index predicts the mortality risk among incident peritoneal dialysis patients in a cohort study. Sci Rep. 2025;15(1):35283. 10.1038/s41598-025-19171-8 PMC12511594 41068227

[5] Ziegler A, et al. Differences in bile acid profiles between cholestatic diseases - Development of a high throughput assay for dried bloodspots. Clin Chim Acta. 2024;562:119864. 10.1016/j.cca.2024.119864 38992821

[6] Yan X, et al. The relationship between bile acid concentration, glucagon-like-peptide 1, fibroblast growth factor 15 and bile acid receptors in rats during progression of glucose intolerance. BMC Endocr Disord. 2017;17(1):60. 10.1186/s12902-017-0211-5 PMC5613331 28946907

[7] Xu H, et al. Gut microbiota-bile acid crosstalk regulates murine lipid metabolism via the intestinal FXR-FGF19 axis in diet-induced humanized dyslipidemia. Microbiome. 2023;11(1):262. 10.1186/s40168-023-01709-5 PMC10675972 38001551

[8] Thomas C, et al. TGR5-mediated bile acid sensing controls glucose homeostasis. Cell Metab. 2009;10(3):167–77. 10.1016/j.cmet.2009.08.001 PMC2739652 19723493

[9] Li B, et al. The relationship between bile acids levels and the prognosis of patients with diabetes on maintenance hemodialysis: a retrospective study. Ren Fail. 2023;45(1):2226221. 10.1080/0886022X.2023.2226221 PMC10286666 37340970

[10] Yang T, et al. A landscape of metabolic variation among clinical outcomes of peritoneal dialysis in end-stage renal disease. Clin Chim Acta. 2024;555:117826. 10.1016/j.cca.2024.117826 38342423

[11] Xiao X, et al. Lower bile acids as an independent risk factor for renal outcomes in patients with type 2 diabetes mellitus and biopsy-proven diabetic kidney disease. Front Endocrinol (Lausanne). 2022;13:1026995. 10.3389/fendo.2022.1026995 PMC9585231 36277729

[12] Levey AS, et al. A new equation to estimate glomerular filtration rate. Ann Intern Med. 2009;150(9):604-612. 10.7326/0003-4819-150-9-200905050-00006 PMC2763564 19414839

[13] Huang L, et al. Nonlinear association between glycated hemoglobin levels and mortality in elderly patients with non-diabetic chronic kidney disease: a national health and nutrition examination survey analysis. Front Endocrinol (Lausanne). 2025;16:1416506. 10.3389/fendo.2025.1416506 PMC11850242 40007805

[14] Liu Y, et al. Farnesoid X receptor agonist decreases lipid accumulation by promoting hepatic fatty acid oxidation in db/db mice. Int J Mol Med. 2018;42(3):1723–31. 10.3892/ijmm.2018.3715 29901078

[15] Clifford BL, et al. FXR activation protects against NAFLD via bile-acid-dependent reductions in lipid absorption. Cell Metab. 2021;33(8):1671–e16844. 10.1016/j.cmet.2021.06.012 PMC8353952 34270928

[16] Tschuck J et al. Farnesoid X receptor activation by bile acids suppresses lipid peroxidation and ferroptosis. Nat Commun. 2023; 14(1). 10.1038/s41467-023-42702-8 PMC10616197 37903763

[17] Wei M, et al. Fibroblast growth factor 15, induced by elevated bile acids, mediates the improvement of hepatic glucose metabolism after sleeve gastrectomy. World J Gastroenterol. 2023;29(21):3280–91. 10.3748/wjg.v29.i21.3280 PMC10292143 37377582

[18] Stefela A, et al. (E)-7-Ethylidene-lithocholic Acid (7-ELCA) Is a Potent Dual Farnesoid X Receptor (FXR) Antagonist and GPBAR1 Agonist Inhibiting FXR-Induced Gene Expression in Hepatocytes and Stimulating Glucagon-like Peptide-1 Secretion From Enteroendocrine Cells. Front Pharmacol. 2021;12:p713149. 10.3389/fphar.2021.713149 PMC8414367 34483922

[19] Bai XP, et al. Influence of ursodeoxycholic acid on blood glucose, insulin and GLP-1 in rats with liver fibrosis induced by bile duct ligation. Diabetol Metab Syndr. 2023;15(1):18. 10.1186/s13098-023-00989-z PMC9930340 36788623

[20] Dong H et al. Imbalance of bile acids metabolism mediated by gut microbiota contributed to metabolic disorders in diabetic model mice. Biology (Basel). 2025;14(3):291. 10.3390/biology14030291 PMC11940414 40136546

[21] Peng YL, et al. Effects of bile acids on the growth, composition and metabolism of gut bacteria. NPJ Biofilms Microbiomes. 2024;10(1):112. 10.1038/s41522-024-00566-w PMC11496524 39438471

[22] Dong H, et al. Imbalance of bile acids metabolism mediated by gut microbiota contributed to metabolic disorders in diabetic model mice. Biology (Basel). 2025;14(3):291. 10.3390/biology14030291 PMC11940414 40136546

[23] de Vries HD, et al. Elevated plasma bile acids coincide with cardiac stress and inflammation in young Cyp2c70(-/-) mice. Pediatr Res. 2025;97(6):2145–52. 10.1038/s41390-024-03596-4 PMC12122362 39358409

[24] Gu JH, et al. Deoxycholic acid and the risk of death and cardiovascular events among patients with advanced chronic kidney disease. J Nephrol. 2025;38(2):799–801. 10.1007/s40620-024-02185-2 39752120

[25] Frazier R, et al. Deoxycholic Acid and Risks of Cardiovascular Events, ESKD, and Mortality in CKD: The CRIC Study. Kidney Med. 2022;4(1):100387. 10.1016/j.xkme.2021.09.004 PMC8767130 35072049

[26] Mafrici B, et al. Amino acid and protein losses in adult patients receiving maintenance dialysis: A literature review. J Ren Nutr. 2026;36(1):30–48. 10.1053/j.jrn.2025.07.012 40759352

[27] Li R, et al. Targeted metabolomics study of serum bile acid profile in patients with end-stage renal disease undergoinghemodialysis. PeerJ. 2019;7:e7145. 10.7717/peerj.7145 PMC6585905 31245185

[28] Clifford BL, et al. FXR activation protects against NAFLD via bile-acid-dependent reductions in lipid absorption. Cell Metab. 2021;33(8):1671–e16844. 10.1016/j.cmet.2021.06.012 PMC8353952 34270928

[29] Peng YL, et al. Effects of bile acids on the growth, composition and metabolism of gut bacteria. NPJ Biofilms Microbiomes. 2024;10(1):p112. 10.1038/s41522-024-00566-w PMC11496524 39438471

[30] Huang Y, et al. Bile acid-induced IRF3 phosphorylation mediates cell death, inflammatory responses, and fibrosis in cholestasis-induced liver and kidney injury via regulation of ZBP1. Hepatology. 2024;79(4):752–67. 10.1097/HEP.0000000000000611 PMC10948324 37725754

[31] Adachi T, et al. The involvement of endoplasmic reticulum stress in bile acid-induced hepatocellular injury. J Clin Biochem Nutr. 2014;54(2):129–35. 10.3164/jcbn.13-46 PMC3947968 24688223

